# Supplementary figures and images for: Calculating site-specific evolutionary rates at the amino-acid or codon level yields similar rate estimates
Source: PeerJ. 2017 May 30;5:e3391. doi: 10.7717/peerj.3391 (PMC5452972; doi:10.7717/peerj.3391)

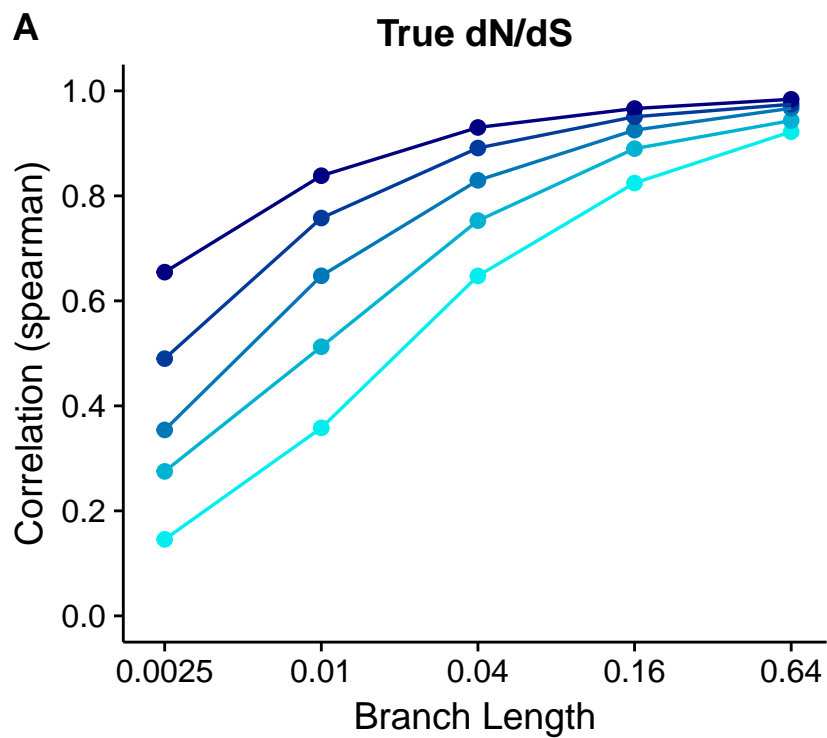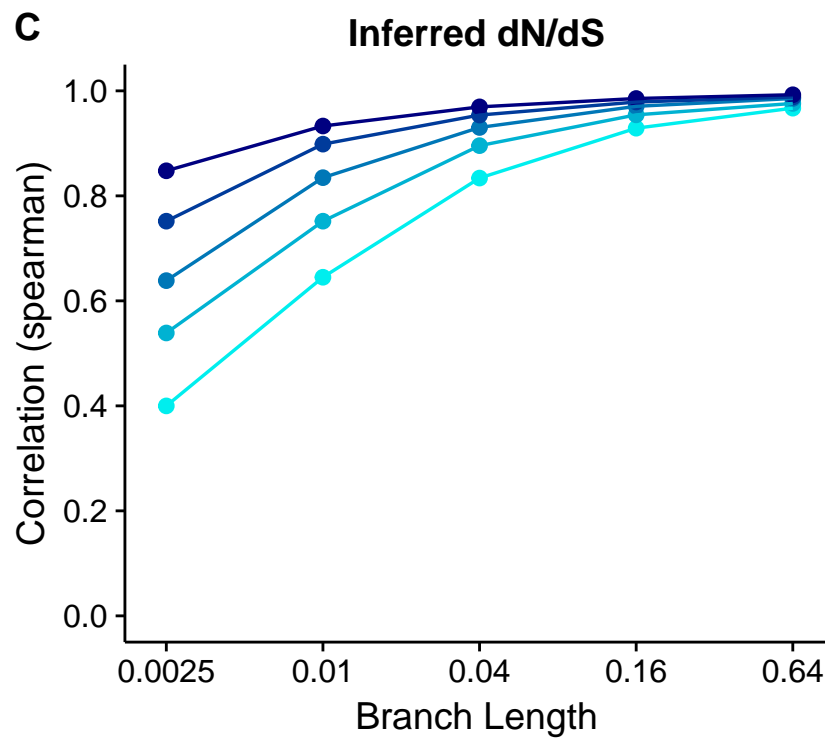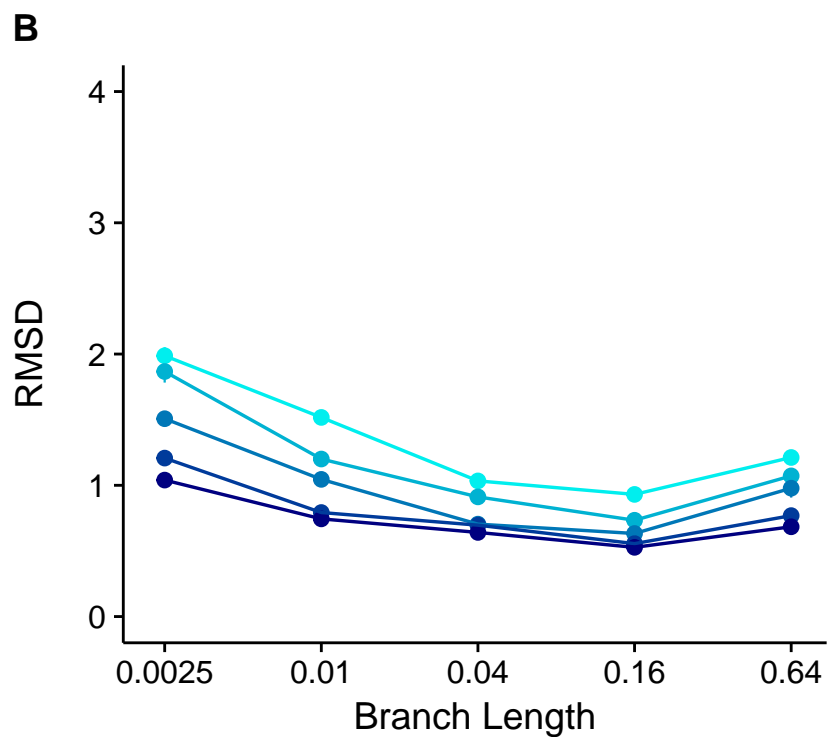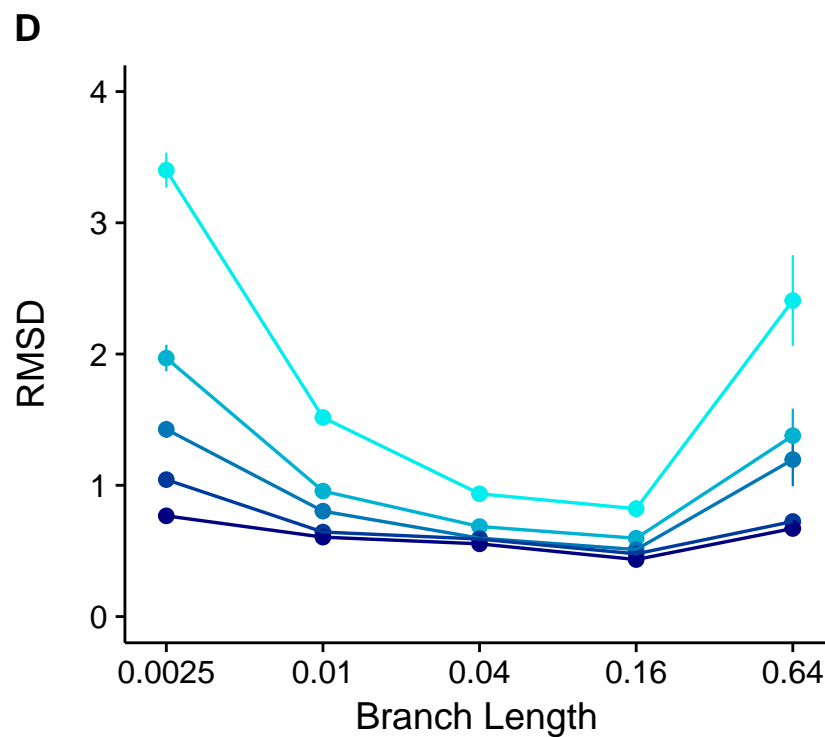

Supplement: Figure S1 — We used the gamma distribution observed for the HIV-1 capsid protein, with shape parameter α = 0.230 and rate parameter β = 0.659 (Meyer & Wilke, 2015b). Each point represents the mean over 50 replicate simulations. The error bars represent the standard error. In nearly all cases, error bars are smaller than the symbol size. (A) Correlations and (B) RMSD values between Rate4Site scores and true dN∕dS. (C) Correlations and (D) RMSD values between Rate4Site scores and inferred dN∕dS. All simulations were performed without codon bias (neutral synonymous codons). [file peerj-05-3391-s001.pdf]

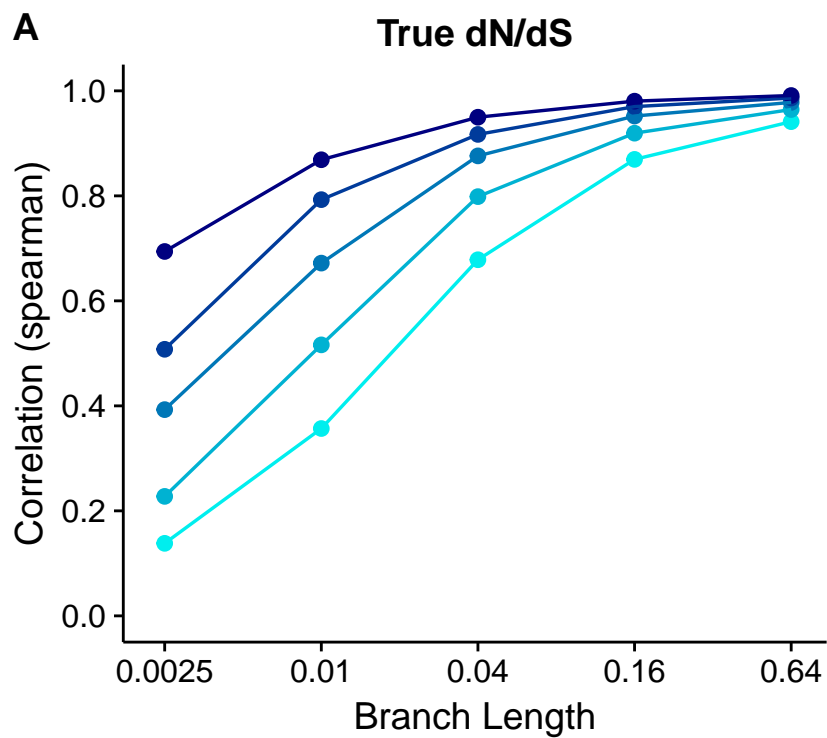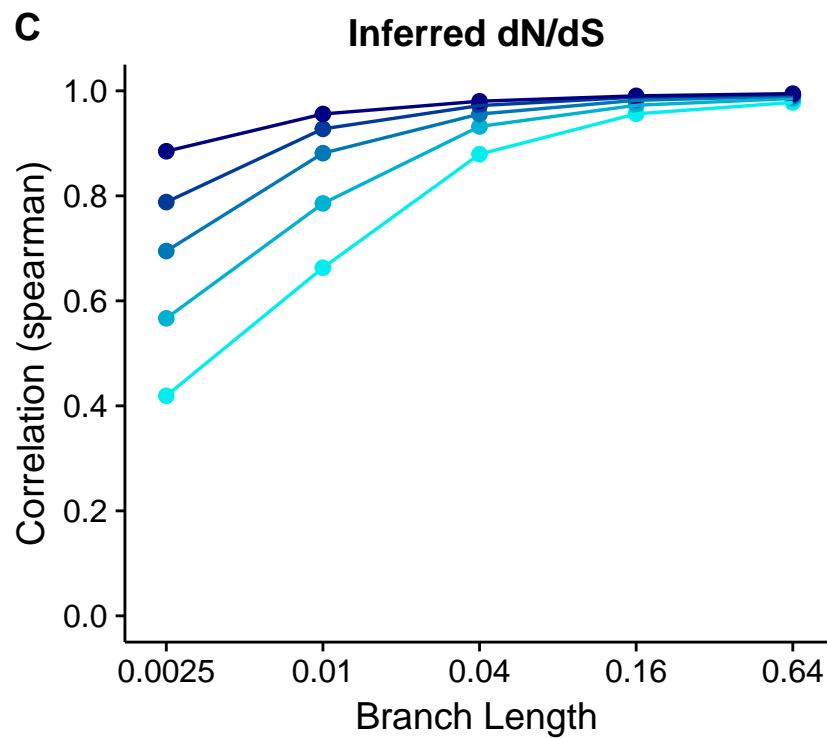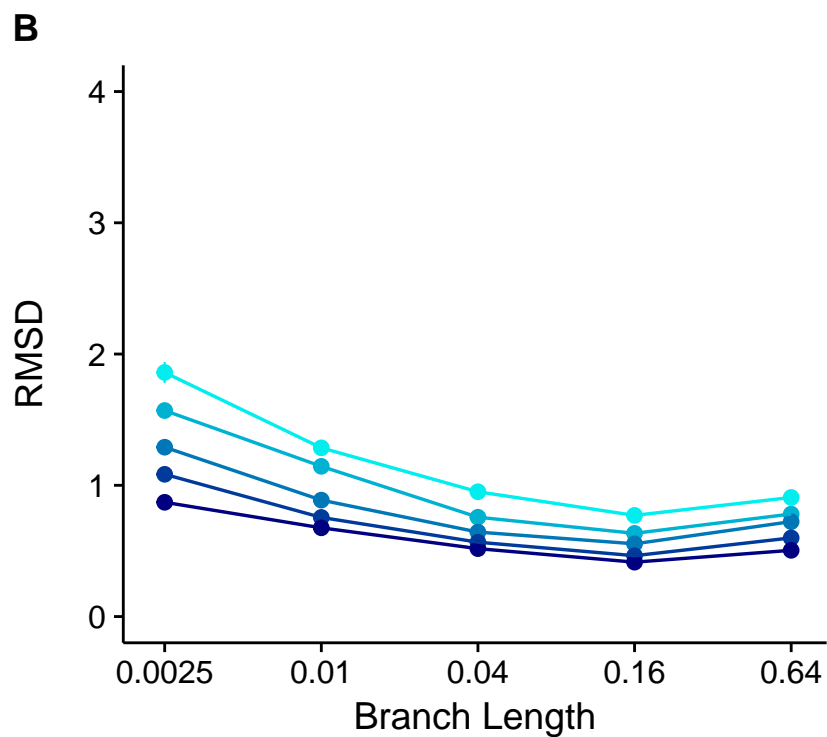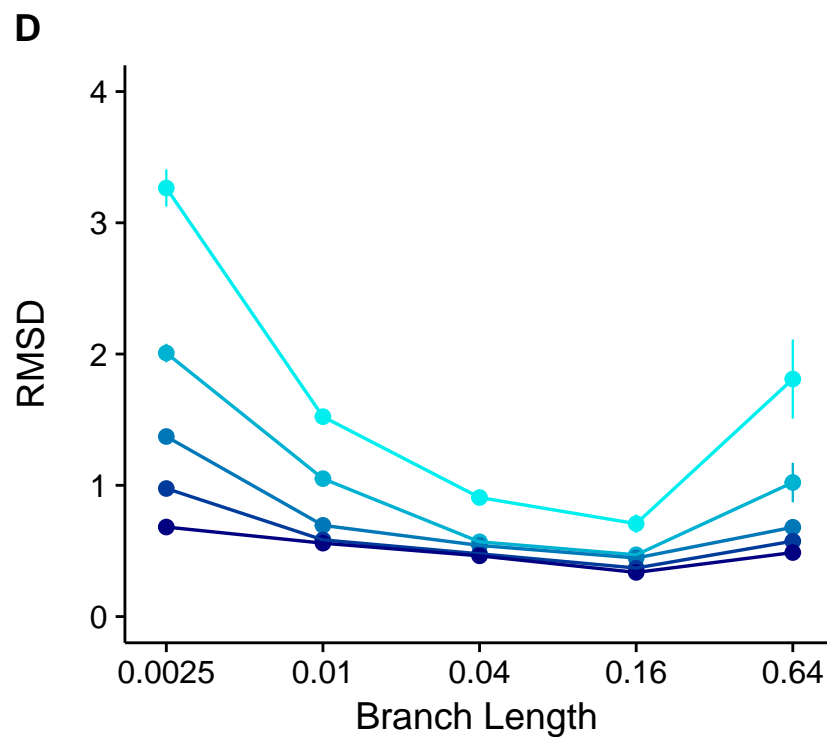

Supplement: Figure S2 — We used the gamma distribution observed for the HIV-1 gp120 protein, with shape parameter α = 0.350 and rate parameter β = 0.249 (Meyer & Wilke, 2015b). Each point represents the mean over 50 replicate simulations. The error bars represent the standard error. In nearly all cases, error bars are smaller than the symbol size. (A) Correlations and (B) RMSD values between Rate4Site scores and true dN∕dS. (C) Correlations and (D) RMSD values between Rate4Site scores and inferred dN∕dS. All simulations were performed without codon bias (neutral synonymous codons). [file peerj-05-3391-s002.pdf]

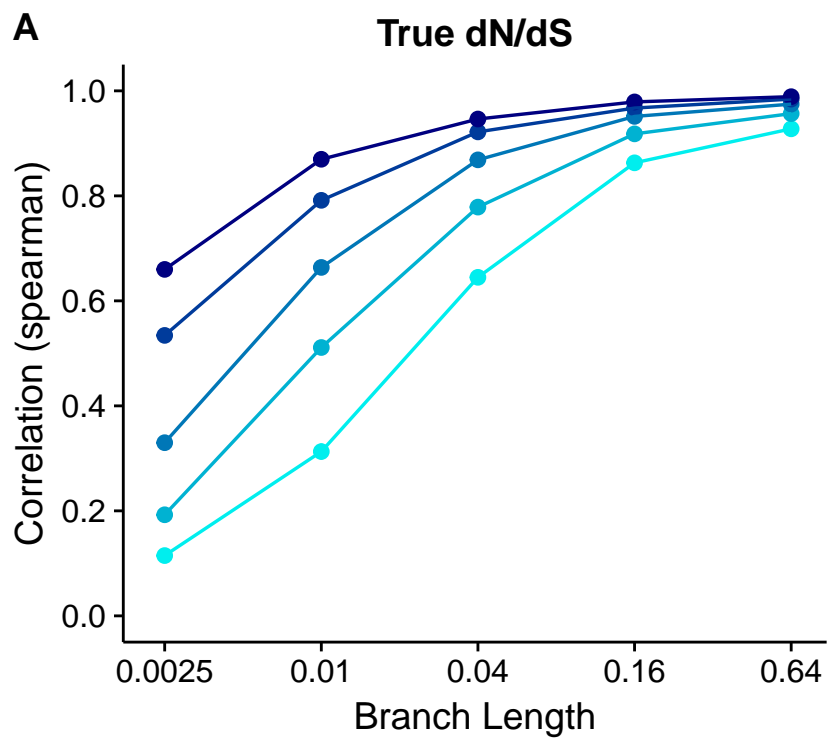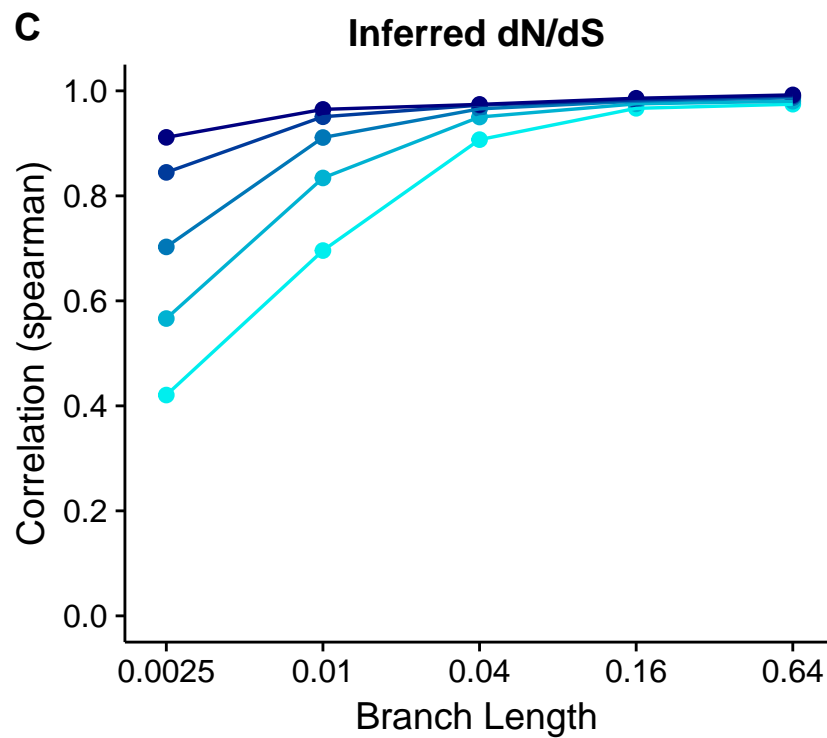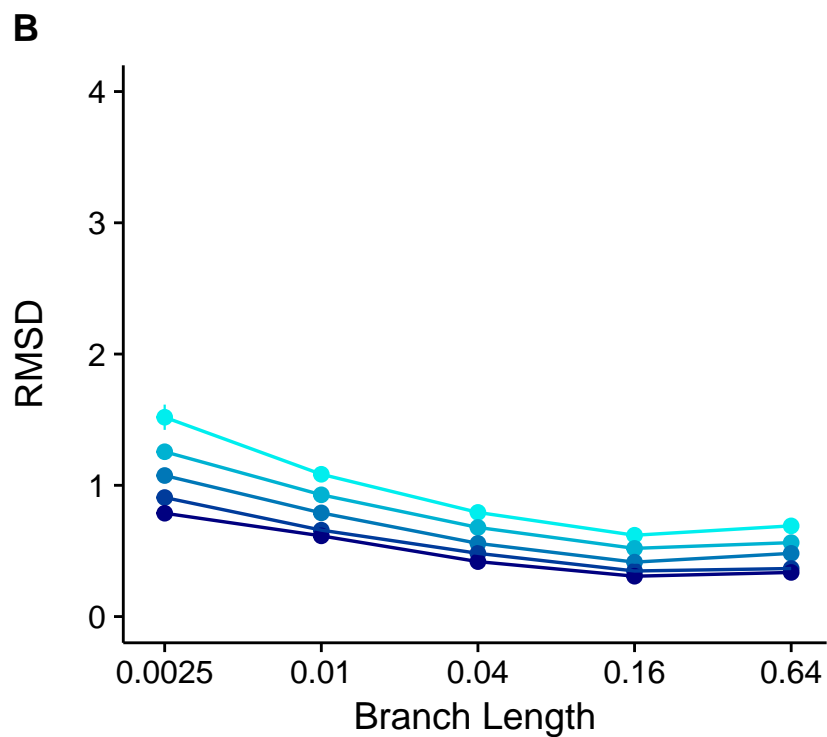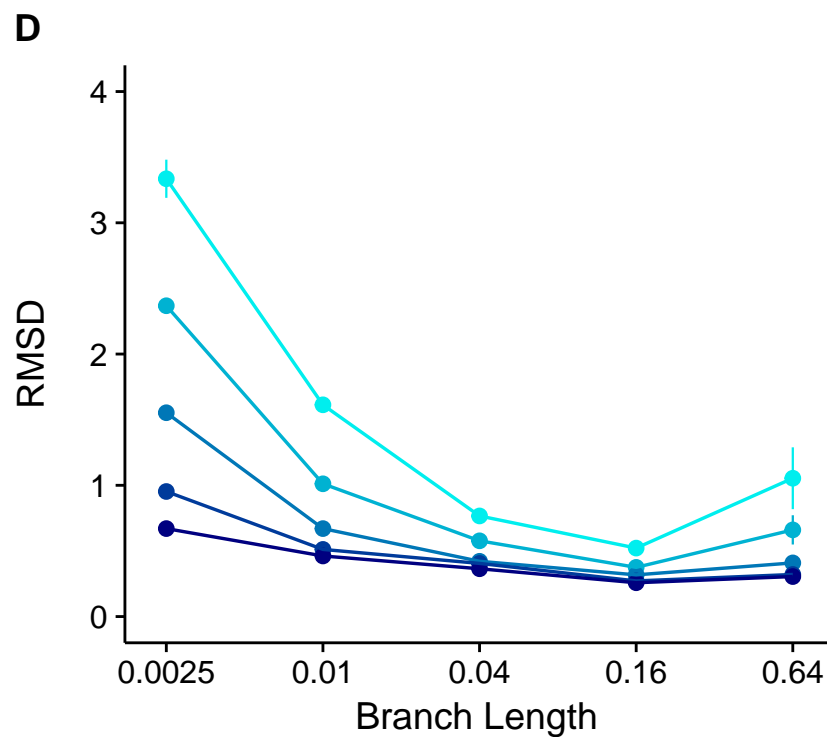

Supplement: Figure S3 — We used the gamma distribution observed for the HIV-1 matrix protein, with shape parameter α = 0.647 and rate parameter β = 0.861 (Meyer & Wilke, 2015b). Each point represents the mean over 50 replicate simulations. The error bars represent the standard error. In nearly all cases, error bars are smaller than the symbol size. (A) Correlations and (B) RMSD values between Rate4Site scores and true dN∕dS. (C) Correlations and (D) RMSD values between Rate4Site scores and inferred dN/dS. All simulations were performed without codon bias (neutral synonymous codons). [file peerj-05-3391-s003.pdf]

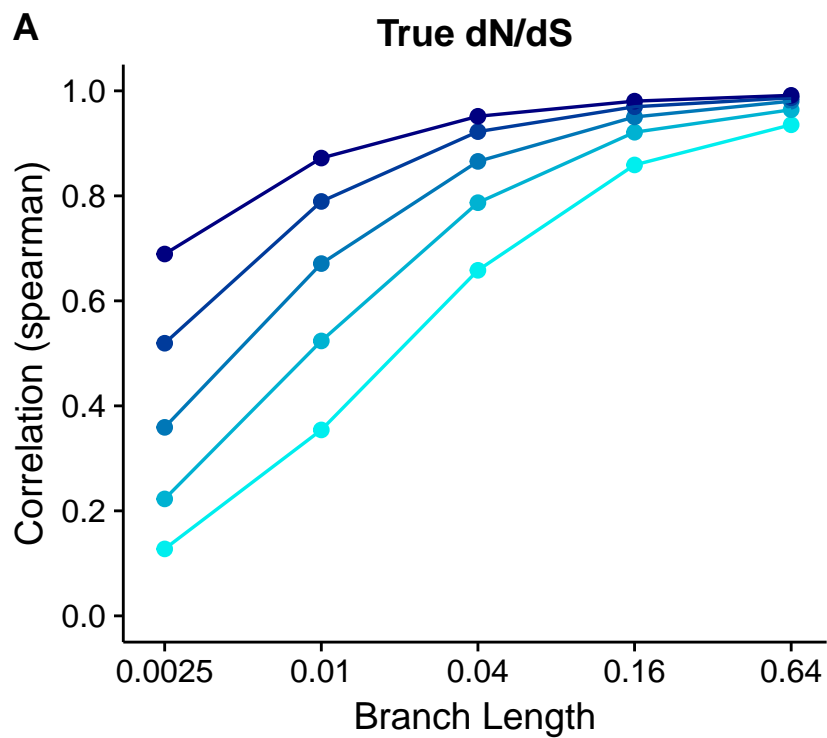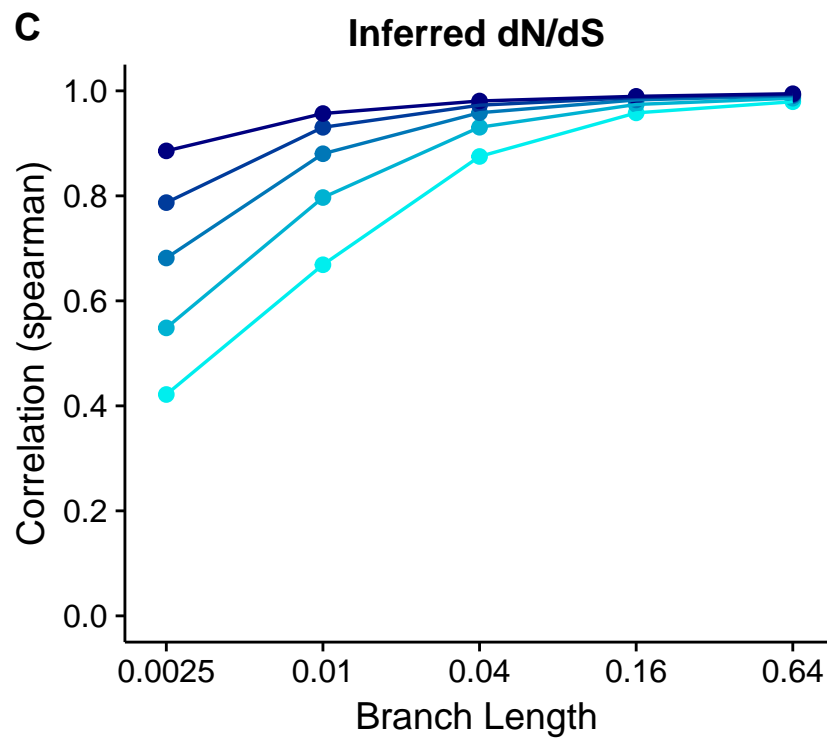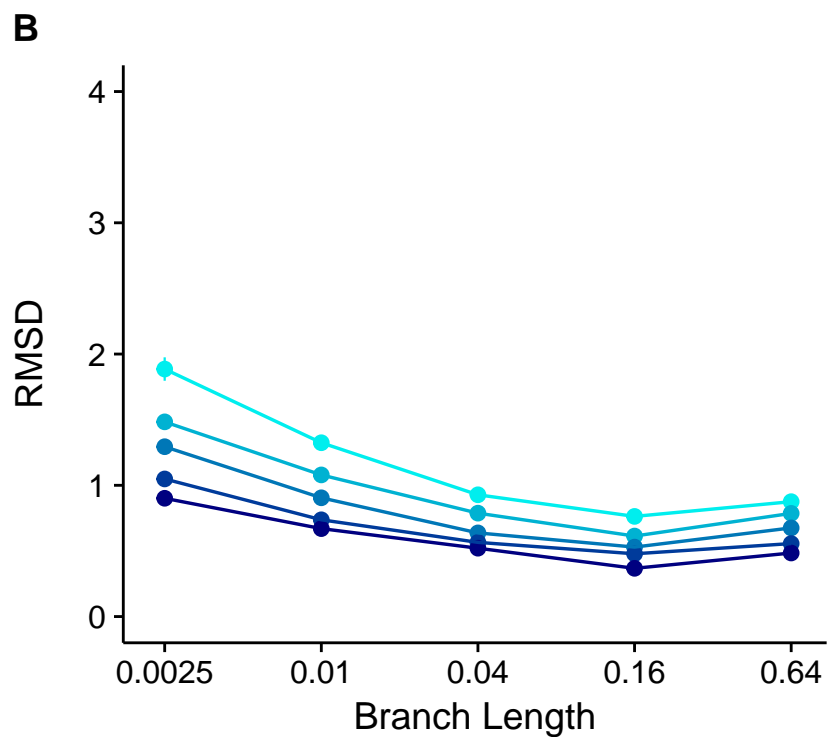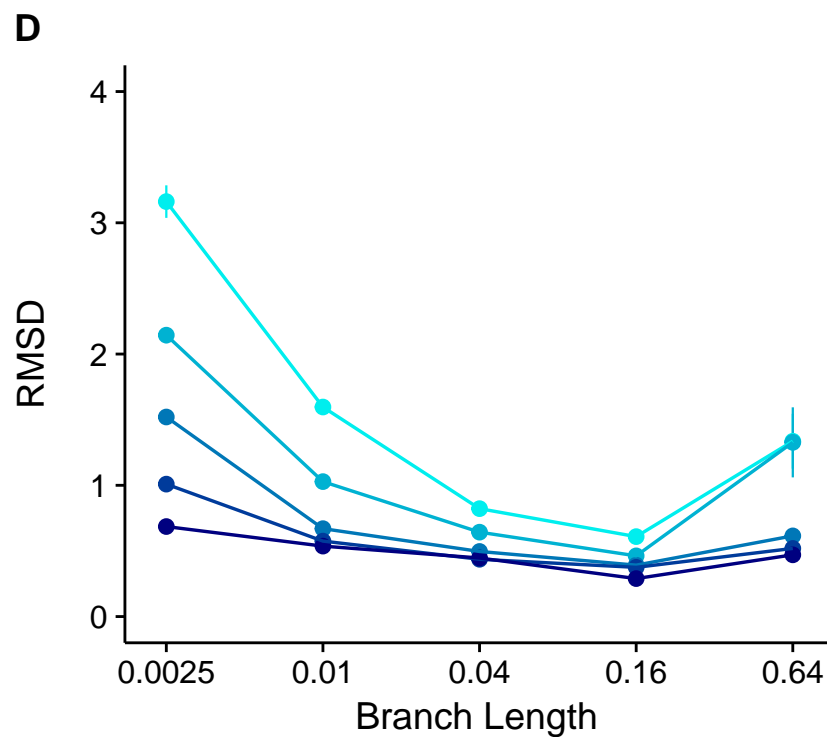

Supplement: Figure S4 — We used the gamma distribution observed for the HIV-1 protease protein, with shape parameter α = 0.378 and rate parameter β = 1.016 (Meyer & Wilke, 2015b). Each point represents the mean over 50 replicate simulations. The error bars represent the standard error. In nearly all cases, error bars are smaller than the symbol size. (A) Correlations and (B) RMSD values between Rate4Site scores and true dN∕dS. (C) Correlations and (D) RMSD values between Rate4Site scores and inferred dN∕dS. All simulations were performed without codon bias (neutral synonymous codons). [file peerj-05-3391-s004.pdf]

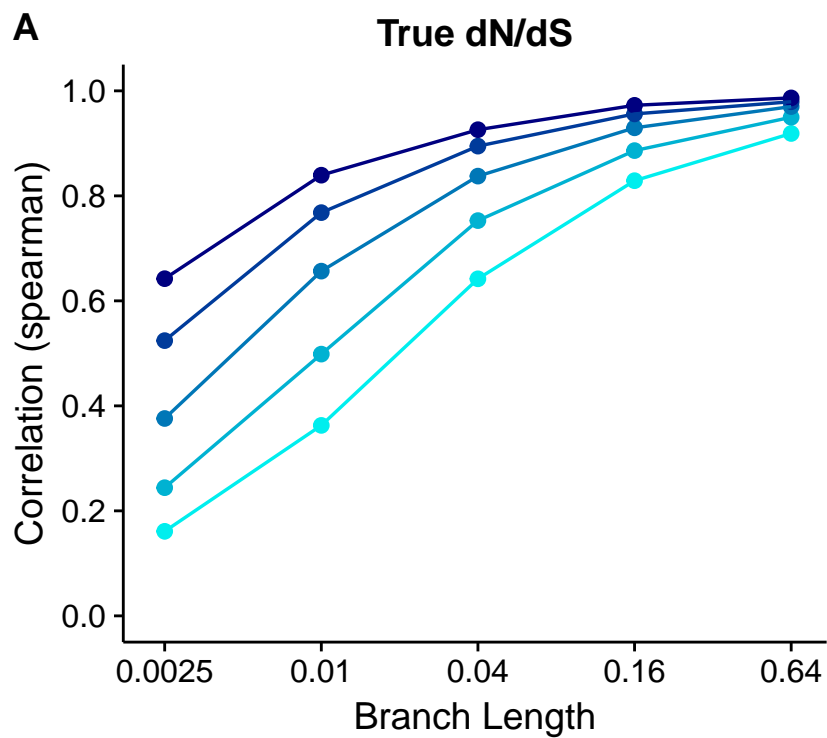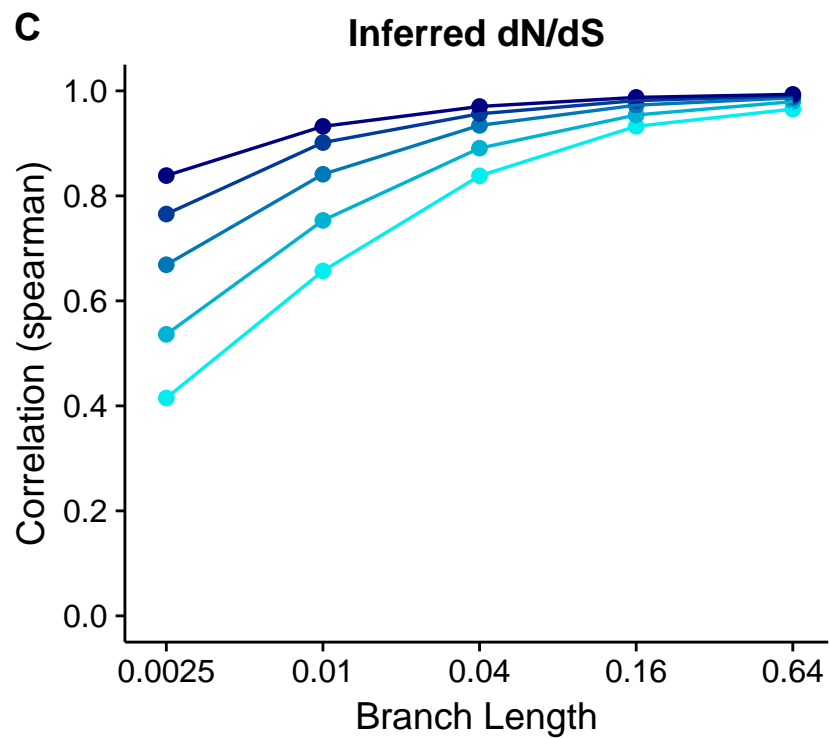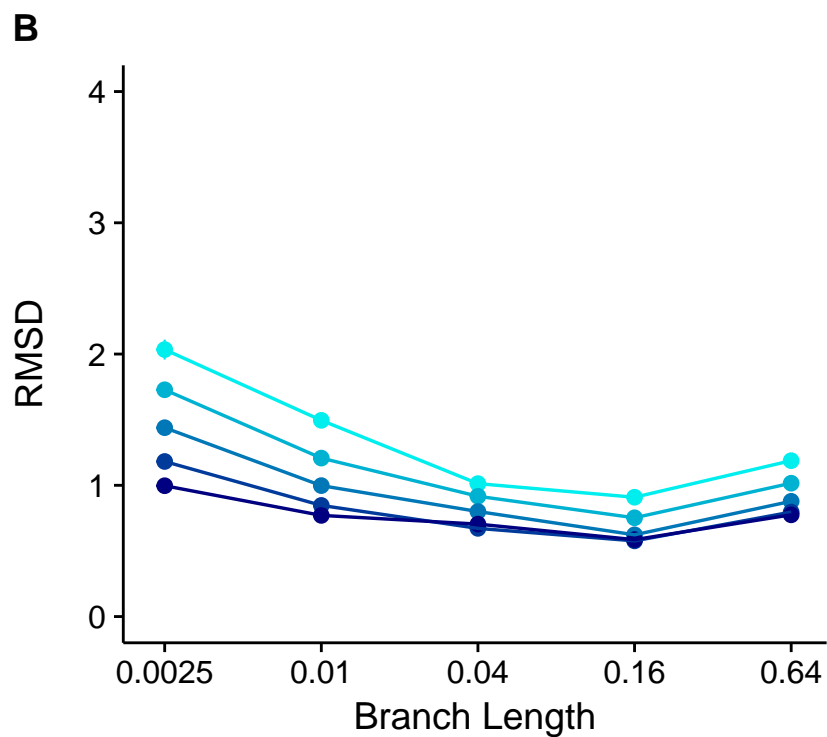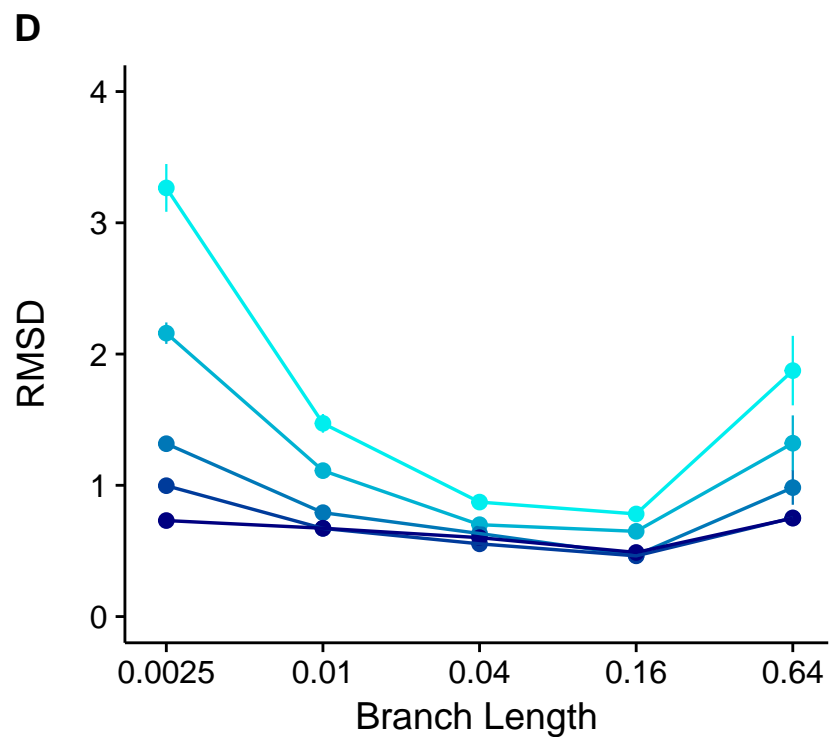

Supplement: Figure S5 — We used the gamma distribution observed for the HIV-1 reverse transcriptase protein, with shape parameter α = 0.238 and rate parameter β = 0.627 (Meyer & Wilke, 2015b). Each point represents the mean over 50 replicate simulations. The error bars represent the standard error. In nearly all cases, error bars are smaller than the symbol size. (A) Correlations and (B) RMSD values between Rate4Site scores and true dN∕dS. (C) Correlations and (D) RMSD values between Rate4Site scores and inferred dN∕dS. All simulations were performed without codon bias (neutral synonymous codons). [file peerj-05-3391-s005.pdf]
